# Atorvastatin as a pleiotropic anticancer agent: mechanisms, evidence, and therapeutic repurposing potential

**DOI:** 10.3389/fimmu.2026.1808729

**Published:** 2026-04-24

**Authors:** Jiaqi Su, Caifeng Ji, Xinlu Niu, Ziming Wu, Lingxue Shi, Longfei Kang, Dongyun Li, Yinling Ma, Guoxun Pang, Xue Ma, Chuan-Min Zhou, Xia Jiang, Bo Pang

**Affiliations:** 1 School of pharmacy, Hebei Medical University, Shijiazhuang, China; 2 Gastrointestinal Disease Diagnosis and Treatment Center, The First Hospital of Hebei Medical University, Shijiazhuang, Hebei, China; 3 Department of General Surgery, Hebei Key Laboratory of Colorectal Cancer Precision Diagnosis and Treatment, The First Hospital of Hebei Medical University, Shijiazhuang, Hebei, China; 4 Department of endocrinology, Shijiazhuang People’s Hospital, Shijiazhuang, Hebei, China; 5 Hebei Key Laboratory of Clinical Pharmacy, Department of Pharmacy, Hebei General Hospital, Shijiazhuang, Hebei, China; 6 Prenatal Diagnosis Center, Shijiazhuang Obstetrics and Gynecology Hospital, Hebei Medical University (Key Laboratory of Maternal and Fetal Medicine of Hebei Province), Shijiazhuang, Hebei, China

**Keywords:** anticancer effects, atorvastatin, cancer therapy, immunomodulation, mevalonate pathway

## Abstract

Cancer remains a leading cause of global mortality, with incidence and mortality rates rising annually. Atorvastatin, a widely used statin, primarily functions by inhibiting 3-hydroxy-3-methylglutaryl-coenzyme A (HMG-CoA) reductase, the rate-limiting enzyme in the mevalonate pathway, thereby lowering cholesterol. Accumulating preclinical and clinical evidence suggests that ATV possesses significant anticancer properties beyond its lipid-lowering effects, positioning it as a promising candidate for adjunctive cancer therapy. The anticancer efficacy of ATV stems fundamentally from its disruption of the mevalonate pathway, which impedes the critical isoprenylation of small GTPases (e.g., Ras, Rho). This inhibition cascades into multifaceted antitumor activities, including the induction of apoptosis and autophagy, dysregulation of the cell cycle, suppression of proliferation, migration, and invasion. ATV further modulates key oncogenic signaling pathways and exhibits potent anti-inflammatory and antioxidant effects within the tumor microenvironment. Crucially, evidence demonstrates that integrating ATV into multimodality regimens—such as alongside immune checkpoint inhibitors and metabolic modulators—significantly improves survival outcomes in patients, substantiating its clinical translational potential. However, a comprehensive and systematic evaluation of its pleiotropic anticancer mechanisms and therapeutic potential is lacking. This review aims to fill this gap by systematically summarizing the efficacy and molecular mechanisms of ATV across various malignancies, alongside its cytoprotective effects on normal tissues. The challenges and future directions for its clinical translation in oncology are also critically discussed.

## 1 Introduction

Despite considerable advances in cancer therapy over the past decades, cancer remains a leading cause of death worldwide, with its global burden continuing to rise (1, 2). Conventional treatment modalities, including surgery, chemotherapy, and radiotherapy, have significantly improved patient outcomes, yet therapeutic resistance and disease recurrence persist as major clinical hurdles (3, 4). This underscores the urgent need for novel strategies, among which drug repurposing has gained prominence for its potential to accelerate oncology drug development. Statins—potent inhibitors of 3-hydroxy-3-methylglutaryl-coenzyme A (HMG-CoA) reductase—have attracted increasing attention in this context, with growing evidence supporting their pleiotropic antitumor effects beyond lipid-lowering (5–8).

Atorvastatin (ATV), one of the most widely prescribed statins, is primarily used for the management of hypercholesterolemia and cardiovascular diseases (9, 10). Its mechanism of action involves competitive inhibition of HMG-CoA reductase, the rate-limiting enzyme in the mevalonate pathway, which is responsible for cholesterol synthesis (3, 11). Beyond lipid-lowering effects, accumulating evidence indicates that ATV exerts pleiotropic actions, including anti-inflammatory, antioxidant, and immunomodulatory activities (12–14). Importantly, preclinical and clinical studies have demonstrated that ATV possesses significant antitumor potential across various cancer types, such as lung, breast, prostate, liver, and colorectal cancers (15–20).

The anticancer effects of ATV are largely attributed to its inhibition of the mevalonate pathway, which disrupts the isoprenylation of small GTPases like Ras and Rho, thereby impairing critical oncogenic signaling cascades such as PI3K/AKT and MAPK pathways (21, 22). This leads to the suppression of tumor cell proliferation, induction of apoptosis and autophagy, and inhibition of metastasis and angiogenesis (23–25). Moreover, ATV has been shown to modulate the tumor immune microenvironment by downregulating PD-L1 expression and enhancing T-cell-mediated antitumor immunity (26, 27). Notably, ATV exhibits a dual role in radiotherapy: it acts as a radiosensitizer in cancer cells while protecting normal tissues from radiation-induced damage (28).

However, a systematic evaluation of ATV’s multifaceted mechanisms, particularly its interplay with the tumor microenvironment and its translational challenges, remains lacking. This review aims to comprehensively summarize the current evidence on the anticancer properties of ATV, elucidate its underlying molecular mechanisms across different cancer types, and critically discuss the obstacles and future directions for its successful integration into oncological therapeutics (**Supplementary Figure 1**).

## 2 Literature search strategy and selection criteria

To ensure a rigorous evaluation of the existing literature, a comprehensive search was conducted exclusively within the PubMed database. The search covered the period from 1990 to 2025, and was restricted to English-language publications. The retrieval strategy utilized a combination of Medical Subject Headings (MeSH) and specific free-text keywords. The primary search string was constructed as follows: “atorvastatin,” “statin,” “cancer,” and “tumor,” combining them in various ways to maximize the comprehensiveness of the search. Examples of these combinations include “atorvastatin AND cancer,” “atorvastatin AND tumor,” “statin AND cancer,” and “statin AND tumor.” These strategies ensure we capture all relevant studies examining the association between atorvastatin and cancer.

### 2.1 Inclusion criteria

(1) Original research articles (*in vitro* studies, *in vivo* animal models, clinical trials, observational studies). (2) Review articles and meta-analyses providing comprehensive mechanistic insights. (3) Studies specifically investigating atorvastatin or statins with particular focus on atorvastatin. (4) Articles examining anticancer mechanisms, therapeutic effects, or chemopreventive effects. (5) Publications in English.

### 2.2 Exclusion criteria

(1) Non-English language publications. (2) Duplicate publications, literature with incomplete clinical research data or obvious errors, or studies with unreasonable experimental design or significant flaws. (3) Publications consisting solely of abstracts.

The retrieved articles were independently screened by the authors to ensure the scientific rigor of the included literature.

## 3 Cholesterol synthesis in malignancies and the significance of atorvastatin

Cholesterol and non-sterol isoprenoids are not merely structural components maintaining membrane integrity, but act as critical mediators that anchor and amplify key oncogenic signaling networks. To satisfy the biosynthetic demands of malignant progression, tumor cells undergo lipid metabolic rewiring, which is the aberrant hyperactivation of the mevalonate pathway to sustain elevated intracellular sterol pools (29).

In this oncological context, to understand the unique significance of ATV, it is necessary to evaluate it alongside other non-statin lipid-lowering agents, such as ezetimibe. Ezetimibe primarily restricts systemic cholesterol availability by targeting the Niemann-Pick C1-Like 1 transporter to block intestinal cholesterol absorption (30). While cholesterol deprivation limits the structural lipids available to tumor cells, ATV displays unique, pleiotropic properties that extend beyond systemic lipid-lowering. By directly inhibiting intracellular HMG-CoA reductase (HMGCR), ATV profoundly truncates the mevalonate pathway (31). This specific blockade depletes key isoprenoid intermediates—such as farnesyl pyrophosphate and geranylgeranyl pyrophosphate—thereby impairing the post-translational prenylation of crucial oncogenic small GTPases, including Ras, Rho, and YAP (31). Consequently, ATV actively disrupts tumor cell signaling, proliferation, and metastasis through intracellular mechanisms that are independent of circulating cholesterol levels. Interestingly, recent real-world clinical cohorts demonstrate that dual metabolic targeting—combining ezetimibe’s absorption blockade with a statin’s intracellular synthesis inhibition—provides a synergistic effect, resulting in a significant reduction in overall cancer risk compared to statin monotherapy (32).

## 4 Targeting tumor cells and the microenvironment: mechanisms of ATV in lung cancer

Lung cancer is the leading cause of cancer-related mortality worldwide. It is pathologically classified into two major subtypes: non-small cell lung cancer (NSCLC) and small cell lung cancer (SCLC). NSCLC, which accounts for the majority of cases, is further subdivided into adenocarcinoma, squamous cell carcinoma, and other histologies (33, 34). Despite advances in conventional chemotherapy, radiotherapy, and targeted therapy, the overall survival for lung cancer patients remains poor, largely due to high rates of recurrence and metastasis (35).

Accumulating evidence suggests that statins, particularly ATV, may hold promise as an adjunctive therapy. Epidemiological studies indicate that long-term statin use is associated with improved survival in NSCLC patients, including those with advanced-stage disease, although no significant benefit has been observed in SCLC (34, 36–41). The antitumor effects of ATV are mediated through multiple interconnected mechanisms. Primarily, by inhibiting the mevalonate pathway, ATV depletes isoprenoids required for the activation of small GTPases (e.g., Rac1), thereby disrupting critical processes for tumor growth and metastasis (42). A key downstream effect is the suppression of Rac1/NADPH oxidase-mediated reactive oxygen species (ROS) production, which leads to the inhibition of vascular endothelial growth factor (VEGF) expression and tumor angiogenesis (42). Additionally, ATV exerts direct effects on cancer cells by inducing cell cycle arrest (e.g., G2/M phase arrest in A549 cells), promoting apoptosis through cholesterol depletion and downregulation of caveolin-1 (Cav1), and inhibiting the pro-metastatic factor MMP-9 (17, 40). These findings are corroborated by *in vivo* studies; for instance, in a benzo(a)pyrene (BaP)-induced lung cancer model, ATV attenuated tumor damage by enhancing endogenous antioxidant enzymes and modulating the expression of apoptotic regulators (e.g., downregulating Bax and caspase-3 while upregulating Bcl-2) (43). In summary, ATV impedes lung cancer progression by simultaneously targeting tumor cells and the tumor microenvironment through the concerted inhibition of the mevalonate pathway and its downstream effectors.

## 5 Multipathway intervention: mechanisms of ATV in hepatocellular carcinoma

Hepatocellular carcinoma (HCC), the most common primary liver malignancy, arises from diverse etiological factors including aflatoxin exposure, chronic heavy alcohol consumption, and hepatitis virus infection (44). While surgical resection remains the cornerstone of treatment for eligible patients, high rates of recurrence and metastasis, often driven by underlying viral oncoproteins and dysregulated signaling pathways, pose major clinical challenges (18). Epidemiological evidence indicates that statins (HMG-CoA reductase inhibitors) may offer chemopreventive benefits against both general and hepatitis B-associated HCC, prompting investigation into their mechanisms of action (45–47).

The antitumor effects of ATV in HCC involve the disruption of multiple oncogenic pathways. Firstly, ATV counteracts key signaling cascades activated by hepatitis virus infection. A prominent mechanism involves the hepatitis B virus X protein (HBx), which promotes metastasis and invasion by activating the PI3K/Akt pathway. ATV antagonizes this oncogenic signal by reducing nuclear pAkt levels, thereby suppressing HBx-driven lipogenesis, invasion, and proliferation through modulation of Gsk3β (48, 49).

Furthermore, ATV effectively disrupts critical autocrine and transcriptional networks that sustain HCC progression. It targets the IL-6/STAT3 autocrine cascade by inhibiting STAT3 phosphorylation, which suppresses the expression of IL-6 and hTERT genes. This leads to reduced IL-6 production and secretion, ultimately restraining cancer cell growth and inducing atypical cellular senescence (50–52). Concurrently, ATV modulates the oncoprotein YAP1, a key driver of cell survival and chemoresistance. By enhancing cisplatin-induced apoptosis through YAP1 downregulation, ATV sensitizes HCC cells to conventional chemotherapy (53–59).

Preclinical evidence robustly demonstrates the tumor-suppressive efficacy of ATV across multiple models. Its activities include: (1) delaying MYC-driven hepatocarcinogenesis in rodents; (2) reducing the growth of xenografts, particularly those derived from HUH-7 cells; and (3) blocking MYC activation via HMG-CoA reductase-dependent pathways in both aflatoxin-induced models and HCC cell lines (60–63).

**Figure 1:**
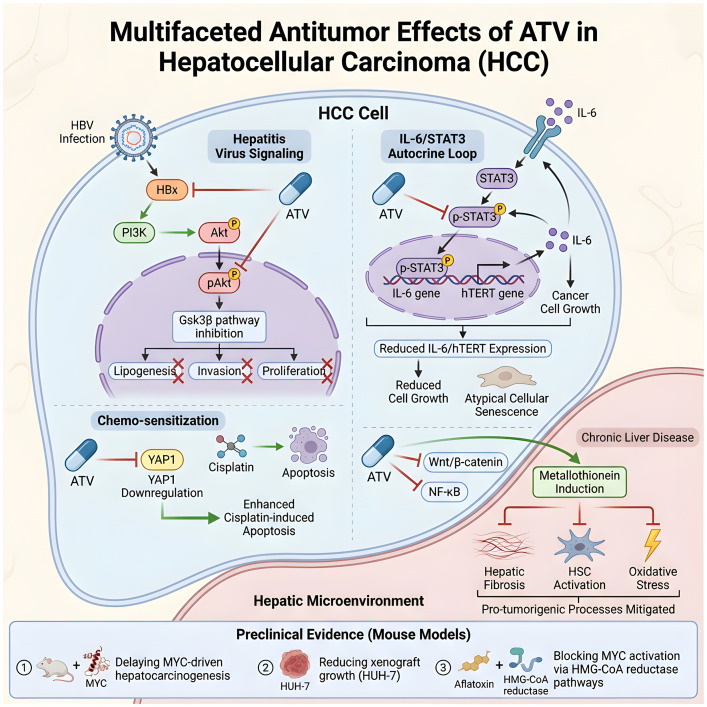
Mechanisms of ATV in hepatocellular carcinoma. ATV counters HBx-driven PI3K/Akt signaling and disrupts the IL-6/STAT3 autocrine loop in HCC cells. It also downregulates YAP1 to enhance chemosensitivity and delays MYC-driven hepatocarcinogenesis.

Beyond targeting neoplastic cells directly, ATV also favorably modulates the hepatic microenvironment to suppress carcinogenesis. It has been shown to prevent HCC development in the context of chronic liver disease by inducing metallothionein expression, an effect mediated through the inhibition of the Wnt/β-catenin and NF-κB pathways (64, 65). Additionally, ATV mitigates key pro-tumorigenic processes in the microenvironment, including hepatic fibrosis, hepatic stellate cell (HSC) activation, and oxidative stress (66). In summary, ATV impedes HCC through a multi-pronged mechanism, simultaneously targeting tumor cells and the supporting stromal microenvironment (**Figure 1**).

## 6 Modulating proliferation and immunity: mechanisms of ATV in breast cancer

Breast cancer represents the second most prevalent malignancy globally (19). Standard treatments include surgery, chemotherapy, radiotherapy, and emerging immunotherapies based on stem cells or dendritic cells (67). Epidemiologic studies have reported an association between lipophilic statin use and reduced breast cancer mortality, and this association is more pronounced in ER/PR-negative subtypes and may be related to inhibition of key proliferative and survival pathways (68, 69).

ATV exerts its effects primarily by inhibiting the mevalonate pathway. The rate-limiting enzyme of this pathway, HMGCR, is often upregulated in breast cancer (19). Through this inhibition, ATV suppresses cancer cell growth by repressing oncogenic YAP signaling and downregulating PDZ-binding kinase (PBK) (70). Additionally, ATV modulates the tumor immune microenvironment by reducing surface PD-L1 expression on cancer cells via mTOR inhibition and by impairing the secretion of PD-L1-carrying extracellular vesicles through disruption of Rab27a isoprenylation. These actions jointly hinder PD-1/PD-L1-mediated T-cell exhaustion and enhance anti-tumor immunity (26).

**Figure 2:**
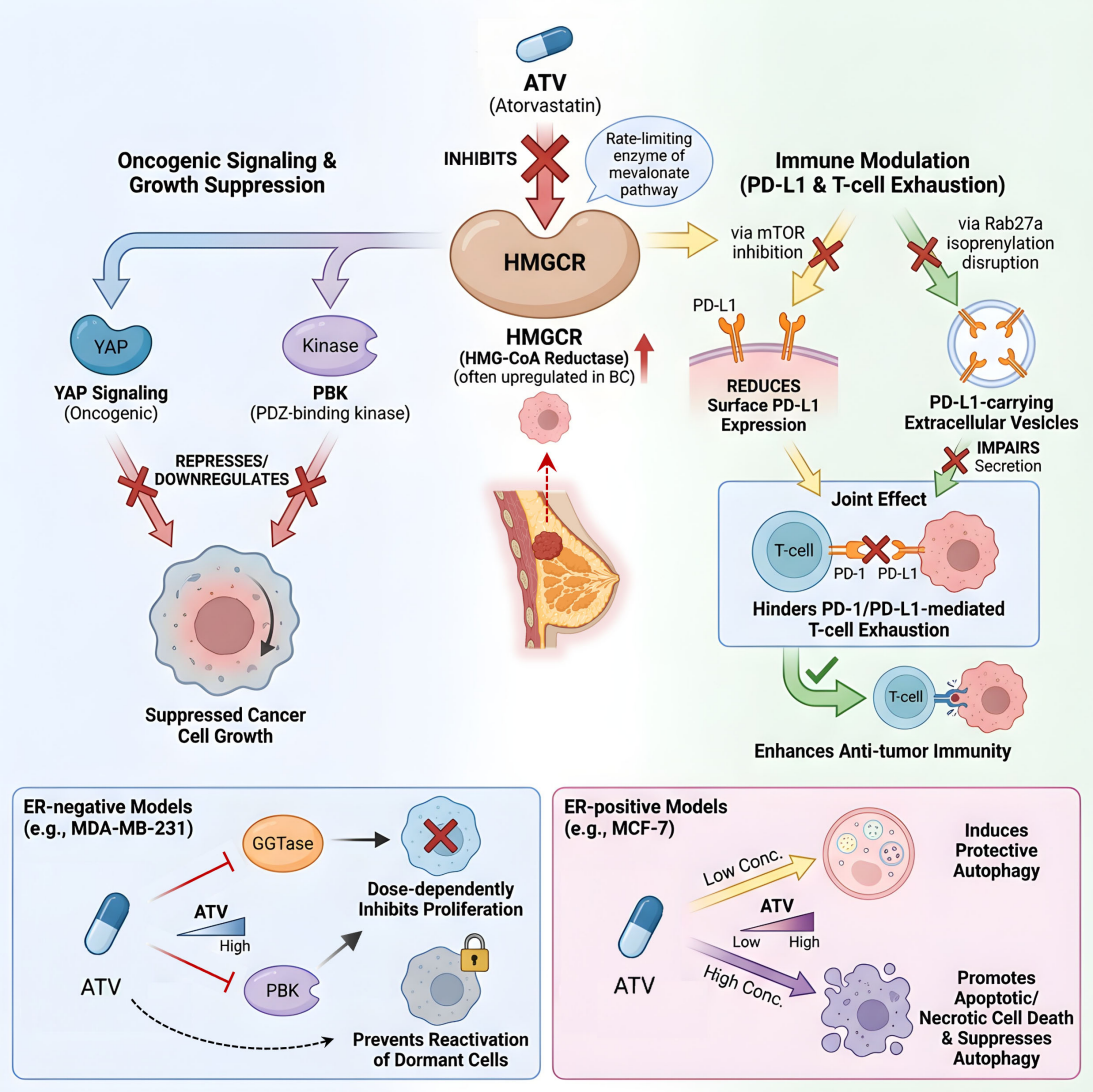
Mechanisms of ATV in breast cancer. ATV inhibits HMGCR, suppressing YAP signaling/PBK and downregulating PD-L1 via mTOR inhibition and Rab27a disruption, enhancing anti-tumor immunity. It shows subtype-specific effects: inhibiting proliferation in ER-negative models and inducing concentration-dependent cell death in ER-positive models.

The anticancer activity of ATV varies across breast cancer subtypes. In ER-negative models such as MDA-MB-231 cells, ATV dose-dependently inhibits proliferation by blocking geranylgeranyltransferase (GGTase) and downregulating PBK, and it can prevent the reactivation of dormant tumor cell subpopulations (70, 71). In ER-positive MCF-7 cells, ATV exhibits a concentration-dependent dual effect: lower concentrations induce protective autophagy, while higher concentrations promote apoptotic or necrotic cell death and concurrently suppress autophagic activity (20, 72, 73) (**Figure 2**).

## 7 From metabolic regulation to radiosensitization: mechanisms of ATV in prostate cancer

Prostate cancer (PCa) represents the most prevalent malignancy among men in most countries and ranks among the top three causes of cancer-related mortality in Western nations (74). Standard management includes radical prostatectomy for localized disease, with endocrine therapy (androgen deprivation therapy, ADT), chemotherapy, and radiotherapy used in advanced stages; however, the development of castration-resistant prostate cancer (CRPC) remains a major therapeutic challenge (75–78). Notably, postoperative biochemical recurrence (BCR) after radical prostatectomy is common. Large database analyses indicate a dose-dependent inverse association between statin use and the risk of BCR, highlighting the potential of statins like ATV as adjunctive agents (79–81).

Building on its canonical mechanism of inhibiting the mevalonate pathway, ATV depletes isoprenoids (e.g., FPP, GGPP) in prostate cancer cells, thereby disrupting the geranylgeranylation and oncogenic signaling of small GTPases such as Ras and Rho. This ultimately suppresses proliferation and induces apoptosis in PCa models (82, 83). Beyond direct pathway inhibition, ATV restricts lipid availability to prostate cancer cells by lowering serum LDL concentrations and specifically downregulating key lipids (e.g., LPC) within prostate tissues. These effects directly impair cancer cell adaptation to hypoxia and increase vulnerability under metabolic stress (84).

ATV further influences critical cellular fate decisions, primarily inducing cytoprotective autophagy over apoptosis in many contexts (85, 86). Mechanistically, by inhibiting protein geranylgeranylation, ATV activates ERK/JNK signaling, leading to transcriptional upregulation of autophagy-related genes (LC3, p62, ULK1) and subsequent initiating autophagy (87). This ATV-induced autophagy contributes to cell death in models like PC3 cells, but androgen-dependent LNCaP cells exhibit relative resistance (88).

ATV also modulates the tumor microenvironment to overcome therapy resistance. It counteracts central tumor hypoxia, a key driver of radioresistance, by suppressing hypoxia-inducible factor-1α (HIF-1α) in a dose-dependent manner. This enhances the radiosensitivity of PCa cells (e.g., PC3) and promotes apoptosis (89, 90). Furthermore, ATV acts as a radiosensitizer by prolonging the cytotoxic effects of radiation-induced ROS. It achieves this by downregulating the expression of ROS-generating enzymes (NOX2/NOX4) and, as evidenced in PC-3 cells, by impairing the activity of the antioxidant enzyme superoxide dismutase (SOD). The resulting perturbation of the intracellular redox balance amplifies radiation-induced DNA damage and enhances tumor cell eradication (28, 90, 91).

## 8 Blocking key pathways and potentiating immunity: synergistic mechanisms of ATV in pancreatic cancer

Pancreatic cancer ranks as the fourth leading cause of global cancer mortality, with rising incidence (92, 93). Its tumorigenesis typically evolves through morphologically defined precursor lesions known as pancreatic intraepithelial neoplasia (PanIN) (94).

**Figure 3:**
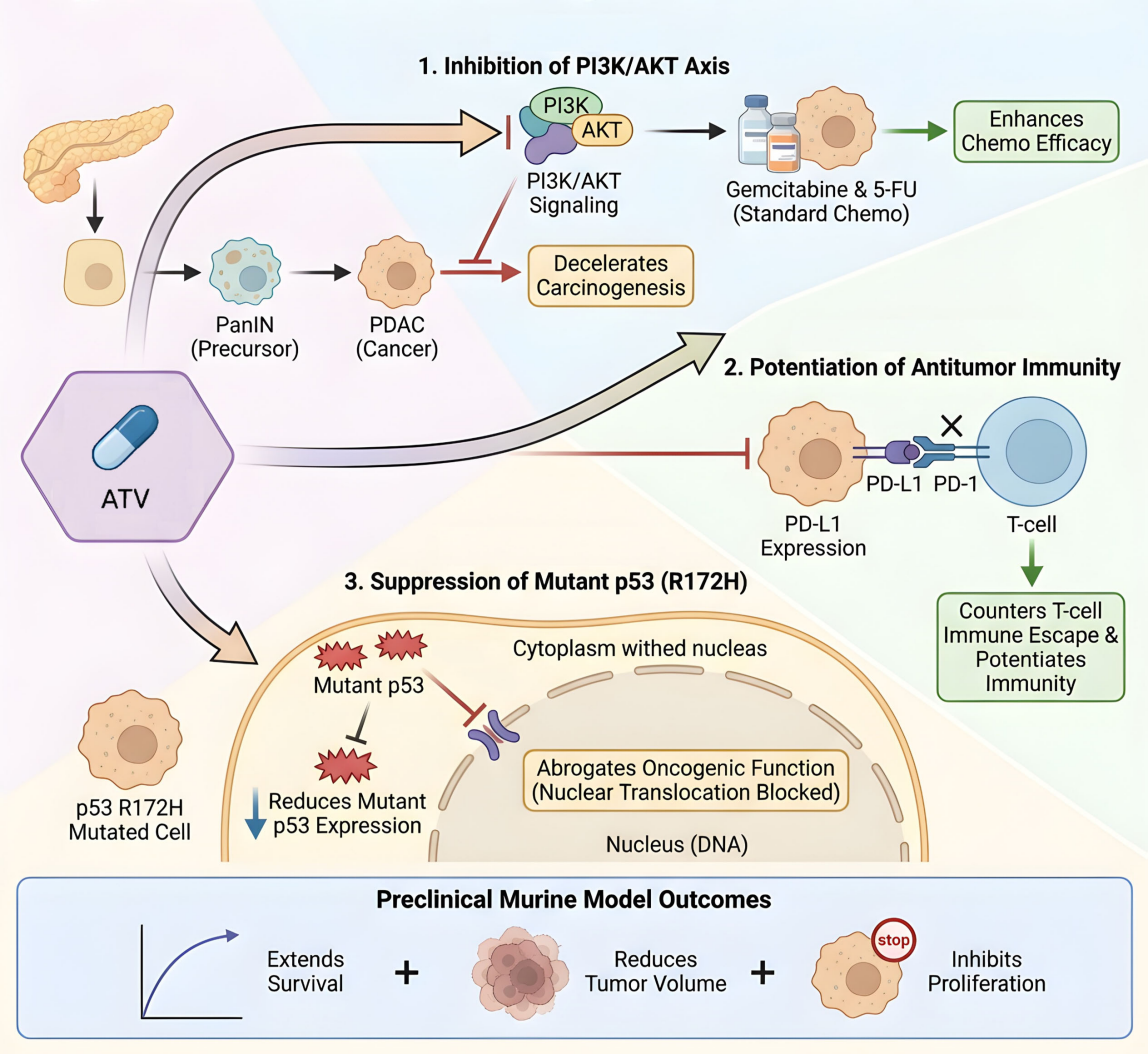
Mechanisms of ATV in pancreatic cancer. ATV inhibits PI3K/AKT signaling, enhances chemo-efficacy, and downregulates PD-L1. In p53-mutant PDAC, it blocks mutant p53 nuclear translocation, suppressing tumor growth *in vivo*.

Clinically, the use of statins such as ATV after diagnosis is associated with reduced mortality in pancreatic cancer patients, highlighting its potential therapeutic value (95). Mechanistically, ATV combats pancreatic cancer through multiple interconnected pathways. A key action is the inhibition of the PI3K/AKT signaling axis, which not only decelerates carcinogenesis from PanIN to pancreatic ductal adenocarcinoma (PDAC) but also enhances the efficacy of standard chemotherapies like gemcitabine and 5-fluorouracil (5-FU) (96–98). Furthermore, ATV potentiates antitumor immunity by downregulating PD-L1 expression, thereby countering T-cell immune escape. These mechanisms are supported by preclinical studies showing that ATV extends survival, reduces tumor volume, and inhibits proliferation in murine models (99). Notably, in PDAC harboring the p53 R172H mutation, ATV suppresses tumor growth and reduces the expression of mutant p53. Crucially, immunofluorescence studies demonstrate that ATV blocks the nuclear translocation of mutant p53, thereby abrogating its oncogenic function (100) (**Figure 3**).

## 9 Concentration-dependent angiomodulation and signaling axis suppression: bidirectional mechanisms of ATV in CRC

Colorectal cancer (CRC) is the third most diagnosed malignancy and the fourth leading cause of cancer death worldwide, with risk influenced by both genetic predisposition and modifiable factors such as obesity (101). Established preventive strategies include screening, lifestyle modification, endoscopic surveillance, and chemoprevention (102). Notably, elevated serum cholesterol is an independent risk factor for CRC, a parameter that can be therapeutically modified by statins (103).

The pleiotropic antitumor effects of statins in CRC are both concentration-dependent and multifaceted. At higher concentrations relevant to antitumor activity, they inhibit tumor angiogenesis, whereas lower concentrations may promote physiological angiogenesis—a duality documented in vascular studies (104, 105).

**Figure 4:**
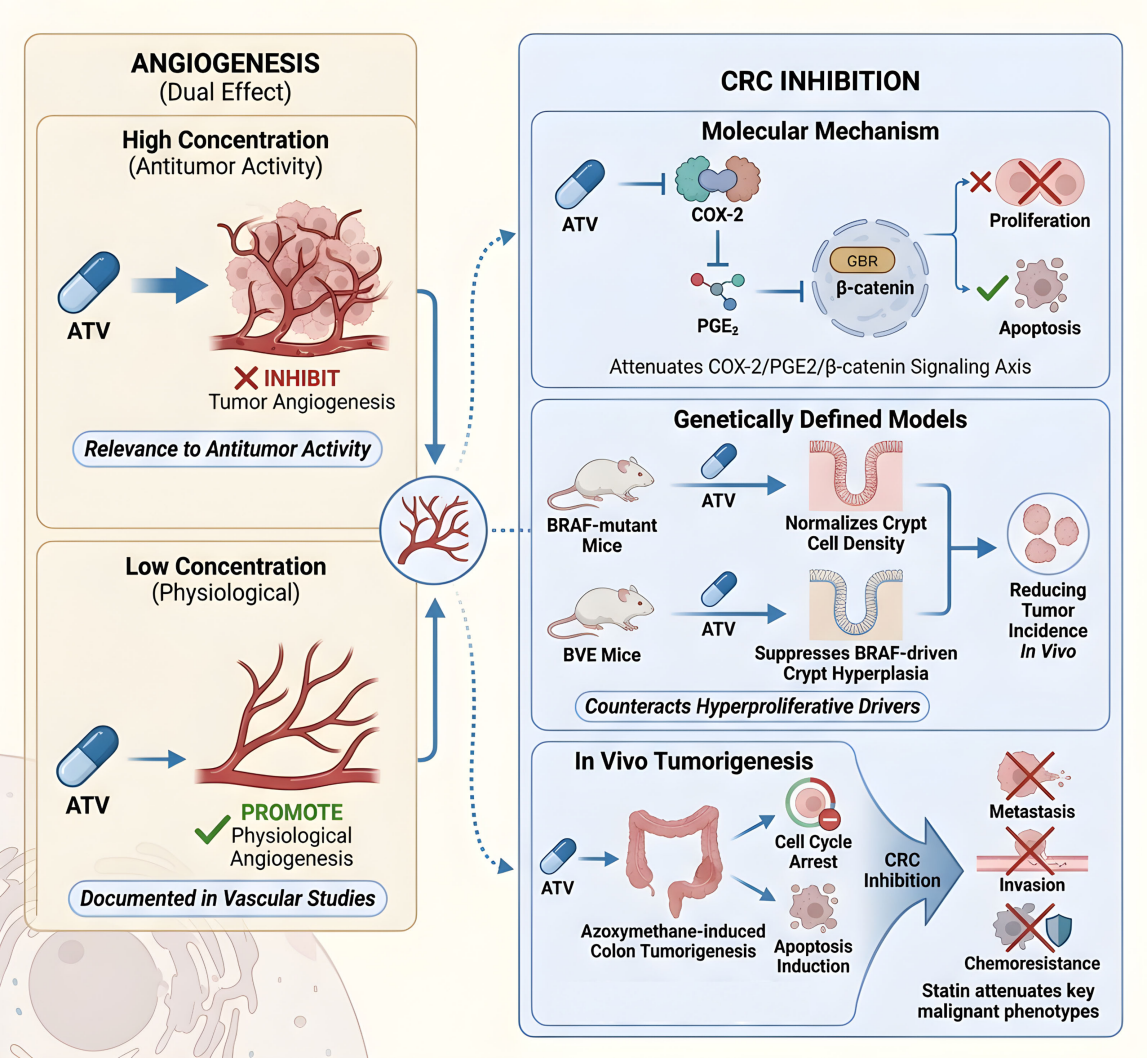
Mechanisms of ATV in colorectal cancer. ATV exhibits concentration-dependent effects on angiogenesis. It inhibits proliferation via the COX-2/PGE2/β-catenin axis and suppresses hyperproliferation in BRAF-mutant models, reducing tumorigenesis.

Mechanistically, ATV disrupts several oncogenic pathways and processes critical for colorectal carcinogenesis. It inhibits proliferation and promotes apoptosis in CRC cells, such as HCT116, by attenuating the COX-2/PGE2/β-catenin signaling axis (106). Furthermore, ATV counteracts hyperproliferative drivers in genetically defined models: it normalizes crypt cell density in BRAF-mutant mice and suppresses BRAF-driven crypt hyperplasia in BMP pathway-competent (BVE) mice, thereby reducing tumor incidence (107). These effects are corroborated by findings that ATV downregulates proliferative gene programs and diminishes overall tumor susceptibility *in vivo (*108). Finally, monotherapy with ATV suppresses azoxymethane-induced colon tumorigenesis through cell cycle arrest and apoptosis induction, while also enhancing cell death in HT29 models and reducing tumorigenicity (109) (**Figure 4**).

## 10 Chemopreventive and therapeutic potential: mechanisms of ATV in bladder cancer

Bladder cancer ranks as the fourth most prevalent malignancy in men and the eighth in women within developed countries, characterized by high incidence and prevalence (110, 111). Established risk factors include occupational exposure to chemicals such as aromatic amines and tobacco use (112). Clinical management is stage-dependent: non-muscle-invasive bladder cancer (NMIBC) is primarily treated with transurethral resection and intravesical chemotherapy, while muscle-invasive bladder cancer (MIBC) typically requires neoadjuvant chemotherapy followed by radical cystectomy (113).

Preclinical studies underscore the chemopreventive and antitumor potential of ATV in bladder cancer. *In vivo*, ATV significantly reduces the incidence and volume of bladder tumors induced by the carcinogen N−butyl−N−(4−hydroxybutyl) nitrosamine (BBN) in rodent models (110). At the cellular level, ATV suppresses proliferation, induces apoptosis, and activates autophagy. Specifically, it triggers autophagic flux in human bladder cancer cell lines (e.g., T24, J82), which correlates with reduced viability and increased apoptotic death (51). Notably, co−treatment with autophagy inhibitors enhances ATV−induced cytotoxicity and apoptosis, suggesting that autophagy may initially serve as a survival mechanism under statin treatment (51, 114).

The antitumor effects of ATV are primarily associated with its ability to inhibit cholesterol biosynthesis. By targeting HMGCR, ATV reduces cellular cholesterol levels, which are often elevated in cancer cells. This effect is further enhanced in the presence of farnesoid X receptor (FXR) overexpression, where ATV acts synergistically with FXR to significantly suppress the cholesterol synthesis pathway through the activation of AMPK and the downregulation of sterol regulatory element-binding protein 2 (SREBP2). As a result, ATV disrupts critical malignant processes, including migration, invasion, and angiogenesis, highlighting its multi−level mode of action in bladder cancer (113).

## 11 Inhibiting invasion and metastasis: mechanisms of ATV in melanoma

Melanoma is an aggressive cutaneous malignancy with a pronounced racial disparity in incidence, being most prevalent among Caucasian populations. Current management strategies extend beyond foundational surgery and adjuvant radiotherapy/chemotherapy to include immunotherapy, targeted therapy (e.g., BRAF/MEK inhibitors), and photodynamic approaches, with immune checkpoint inhibitors and kinase-targeted therapies constituting the modern standard of care (115). Metastatic disease is notably aggressive and often resistant to conventional treatments (116). Given this clinical challenge, modulating cholesterol metabolism has emerged as a potential strategy to impede tumor progression, enhance chemotherapy efficacy, and provide chemopreventive benefits (117, 118).

**Figure 5:**
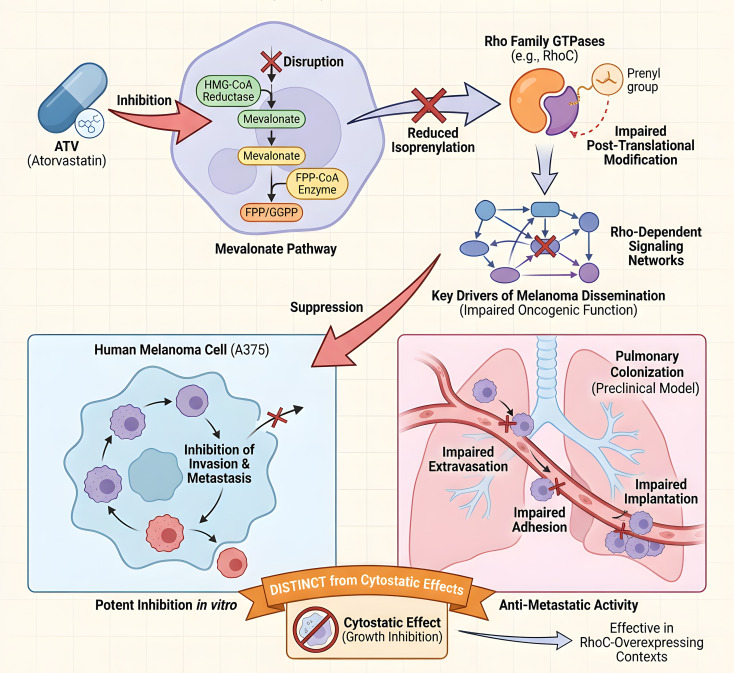
Mechanisms of ATV in melanoma. By disrupting the mevalonate pathway, ATV impairs isoprenylation of Rho GTPases (e.g., RhoC). This suppresses Rho-dependent signaling, inhibiting tumor cell invasion, adhesion, and pulmonary colonization.

Accumulating evidence supports the anti-melanoma properties of statins, particularly ATV. The efficacy of ATV in melanoma is largely attributed to its disruption of the mevalonate pathway, which critically impairs the post-translational modification (isoprenylation) and oncogenic function of Rho family GTPases—key drivers of melanoma dissemination (119). Consequently, ATV potently inhibits invasion and metastasis in human melanoma cells (e.g., A375) through the suppression of Rho-dependent signaling networks (120). Preclinically, ATV’s anti-metastatic activity is evidenced by its inhibition of pulmonary colonization, a process involving the impairment of tumor cell extravasation, adhesion, and implantation, distinct from cytostatic effects. This activity extends to various molecular contexts, including RhoC-overexpressing melanomas (115, 116) (**Figure 5**).

## 12 Multimodal antitumor activity: mechanisms of ATV in glioblastoma

Glioblastoma multiforme (GBM) is the most common and aggressive primary malignant brain tumor, characterized by diffuse infiltration, therapeutic resistance, and a poor prognosis. Its progression is driven by a complex tumor microenvironment that promotes gliomagenesis, angiogenesis, and treatment evasion (121–123). A key adaptation within this microenvironment is intense neovascularization, largely mediated by hypoxia-induced upregulation of vascular endothelial growth factor-A (VEGF-A) (122).

Given the central role of dysregulated signaling and angiogenesis in GBM, strategies to modulate these pathways are of high therapeutic interest. Preclinical studies consistently demonstrate the anti-glioblastoma activity of ATV, which acts through multiple mechanisms targeting both the tumor microenvironment and tumor cells (124–127). ATV inhibits the mevalonate pathway, leading to reduced membrane localization of epidermal growth factor receptor (EGFR) and subsequent suppression of EGFR/AKT signaling, thereby impairing energy metabolism and proliferation, and enhancing the efficacy of temozolomide (128). Furthermore, by inhibiting cholesterol biosynthesis, ATV disrupts lipid metabolism pathways that are particularly crucial in 6-O-Methylguanine-DNA Methyltransferase (MGMT) promoter unmethylated glioblastoma (126). ATV also exerts potent anti-angiogenic effects by downregulating VEGF expression. Additionally, it suppresses glioma cell invasion and promotes apoptosis through modulation of apoptosis regulators (e.g., caspase-3, Bcl-2) and downregulation of the chemokine receptor CXCR4 (122, 129, 130). These multi-targeted actions collectively inhibit proliferation and induce apoptotic cell death, as evidenced in both conventional and 3D glioma spheroid models.

## 13 Antitumor and antimetastatic actions: mechanisms of ATV in ovarian cancer

Ovarian cancer is the leading cause of mortality among gynecological malignancies (131, 132). This lethal disease predominantly presents at advanced stages, contributing to its characteristically poor prognosis (131, 133, 134). Epidemiological studies have linked long-term statin use to a reduced incidence of ovarian cancer, highlighting the potential relevance of perturbed lipid metabolism and inflammation in its pathogenesis (131).

The biological rationale for this association is rooted in key oncogenic pathways. Malignant progression in ovarian cancer is supported by the activation of the mevalonate pathway, which furnishes essential lipid components for proliferation and survival (135, 136). Concurrently, a pro-inflammatory tumor microenvironment, characterized by elevated cytokines such as IL-6, TGF-β1, and TNF-α, promotes angiogenesis and metastasis via factors like VEGF and IL-8 (137, 138). Preclinical studies demonstrate that statins can directly modulate this milieu by significantly reducing the levels of these pro-tumorigenic cytokines in ovarian cancer cell lines (131). Mevalonate pathway inhibitors, therefore, exert multifaceted antitumor effects, including apoptosis induction and the suppression of metastasis and invasion (29, 131, 139).

ATV exerts direct antitumor effects through multiple interconnected mechanisms. It inhibits ovarian cancer cell viability by inducing G1-phase cell cycle arrest, promoting apoptosis, and activating autophagic flux. This ATV-induced cellular stress triggers reactive oxygen species (ROS) overproduction and endoplasmic reticulum stress, leading to the upregulation of autophagy-related proteins (e.g., ATG3, Beclin-1) and the initiation of mitochondrial apoptosis. Furthermore, ATV potently suppresses the migratory and invasive capacities of ovarian cancer cells in a dose-dependent manner, thereby impeding critical steps in metastasis (136).

## 14 Discussion

### 14.1 Controversies and heterogeneous effects

The anticancer potential of statins, particularly ATV, represents a dynamic and evolving area of oncological research, with evidence accumulating across diverse cancer types and molecular pathways (3) (**Supplementary Table 1**). The principal anticancer mechanisms encompass the inhibition of tumor cell proliferation and the induction of cell death. Nonetheless, the clinical and preclinical data pertaining to ATV are characterized by controversy and heterogeneity.

Evidence regarding the cancer-preventive and therapeutic efficacy of ATV remains inconsistent across different malignancies. While preclinical models, such as chemically-induced bladder cancer, demonstrate clear antitumor effects for ATV (114), population-based studies have not yet established a definitive causal relationship for risk reduction in humans. Similarly, epidemiological findings on statin use and prostate cancer risk are conflicting, showing either a reduced risk or neutral effects, discrepancies that may be attributed to biases in PSA screening or variations in statin dosage and type (140, 141). Although the anticancer potential of ATV is supported by substantial evidence, its efficacy frequently exhibits mechanistic contradictions modulated by metabolic state, dose, and tumor heterogeneity. Recent evidence reveals that baseline metabolic states determine ATV’s efficacy in HCC. Inhibiting cholesterol *de novo* synthesis under high fatty acid diets paradoxically promotes HCC progression by activating SREBP2-mediated Prostaglandin E Synthase 2 (PTGES2) transcription. In a lipid-rich environment, upregulated PTGES2 utilizes linoleic acid (LA) to produce PGE2, driving HCC proliferation. Crucially, without LA, ATV instead suppresses migration, highlighting arachidonic acid metabolism as a context-dependent variable (142).

Furthermore, ATV’s pleiotropic effects are driven by dose, temporal dynamics, and cellular plasticity. *In vivo* studies reveal that low doses of ATV stimulate melanoma growth and angiogenesis (143), contrasting with its anti-angiogenic properties at higher concentrations in CRC (104, 105). Temporally, while short-term intervention suppresses early lung adenomas, prolonged treatment drives resistance and invasive progression (144). Additionally, statin-induced cholesterol inhibition can induce partial epithelial-to-mesenchymal transition, paradoxically enhancing metastatic seeding in solid tumors (145). Finally, heterogeneity drives discordant outcomes across subtypes; ATV strictly inhibits proliferation in ER-negative breast cancer (70, 71), but induces protective autophagy at lower concentrations in ER-positive cells (20, 72, 73).

### 14.2 Pleiotropic cytoprotection and dual roles in radiotherapy

A significant and distinguishing feature of ATV is its pleiotropic cytoprotective effect on normal tissues, which may help widen the therapeutic window in oncology. It exhibits notable radioprotective and chemoprotective properties, supported by substantial preclinical evidence. For instance, in patients with lung cancer, statins protect normal lung tissue from radiation-induced damage while concurrently exerting anticancer effects (146). ATV also mitigates common chemotherapy-related side effects, such as nausea and fatigue, suggesting a broader role in supportive care (43).

This radioprotective effect is well-substantiated across multiple organ systems. At the cellular level, ATV protects irradiated human umbilical vein endothelial cells and significantly reduces micronucleus formation in irradiated human lymphocytes, indicating reduced genotoxicity (147). *In vivo*, ATV administration protects mouse testicular epithelial cells from radiation damage, attenuates renal tubular injury, and alleviates radiation enteropathy—partly through downregulation of plasminogen activator inhibitor-1 (PAI-1) (148). The underlying mechanisms are multifaceted, involving anti-inflammatory and antioxidant actions, inhibition of lipid peroxidation, enhancement of endogenous defenses, and suppression of pro-apoptotic signaling (e.g., caspase-3 activation) (3). These protective pathways also extend to non-radiation contexts, such as reducing cardiac fibrosis and neuronal apoptosis via modulation of AKT, JNK, and ERK pathways, underscoring ATV’s broad tissue-protective capacity (3, 28, 147, 149). Notably, in certain tumor contexts like PC-3 prostate cancer cells, ATV can also modulate radiation-induced reactive oxygen species (ROS), highlighting its dual role as both a normal tissue protector and a tumor radiosensitizer (28, 147).

### 14.3 Translational challenges and preclinical synergies

Despite compelling preclinical data, the clinical translation of ATV as an anticancer agent faces several challenges. These include optimizing its bioavailability within the tumor microenvironment, defining synergistic dosing regimens with conventional therapies (chemotherapy, radiotherapy), and carefully understanding context-dependent metabolic variables. Advanced delivery systems, such as ATV-loaded micro-nanoparticles for inhalation in lung cancer, are under investigation to improve targeting and efficacy (150).

Beyond monotherapy, ATV shows promise in combination regimens. *In vitro* and *in vivo* studies indicate synergistic antitumor activity when ATV is combined with other agents, such as celecoxib. This combination synergistically inhibited growth and induced apoptosis in PC-3 prostate cancer cells and suppressed tumor growth in immunodeficient mice more effectively than high-dose monotherapies (52). Comparative analyses among statins further suggest that ATV possesses superior *in vitro* potency against several cancer types (e.g., breast cancer, melanoma, glioblastoma, prostate cancer) compared to others like pravastatin (3).

### 14.4 ATV resistance mechanisms and viable countermeasures

Despite ATV’s promising anticancer potential, translating its efficacy into clinical applications is commonly hindered by acquired or intrinsic drug resistance. A primary mechanism of statin resistance involves target-level adaptations, notably the compensatory induction or amplification of the HMGCR gene. Upon ATV exposure, resistant tumor cells activate sterol regulatory element-binding proteins (SREBPs), triggering a massive upregulation of HMGCR to rescue the truncated mevalonate pathway (151). To disrupt this positive feedback loop, preclinical models demonstrate that the concomitant attenuation of HMGCR—either through RNA interference or combined pharmacological degradation—completely abolishes this adaptive resistance and significantly potentiates ATV-mediated growth inhibition (152).

Furthermore, resistant malignancies frequently deploy broad metabolic rewiring to bypass disrupted cholesterol synthesis. For instance, insensitivity to ATV is strongly associated with an accelerated accumulation of intracellular lipid droplets and a substantial increase in exogenous fatty acid metabolism. This metabolic plasticity allows cancer cells to maintain necessary structural lipids despite mevalonate deprivation. Consequently, deploying lipid metabolism inhibitors to co-target fatty acid uptake or beta-oxidation serves as a highly viable countermeasure to eliminate this escape mechanism (153, 154).

Finally, metabolic compensation extends to redox homeostasis. While the truncation of the mevalonate pathway induces severe oxidative stress in tumor cells, resistant clones compensate by upregulating antioxidant metabolic networks, including augmented reductive carboxylation and enhanced cystine import. Crucially, pharmacologically targeting these specific antioxidant responses effectively synergizes with statin-mediated metabolic stress, producing a profound synthetic lethality in resistant tumors (155).

### 14.5 Clinical evidence of ATV-based combination therapies

Although preclinical models provide critical mechanistic insights, translating the anticancer potential of ATV requires robust clinical validation. Recent clinical trials and large-scale cohort studies have significantly bolstered the translational value of ATV, particularly when applied in combination regimens.

Combination with Immune Checkpoint Inhibitors (ICIs): Real-world clinical evidence strongly supports the synergistic potential between statins and ICIs. Comprehensive clinical analyses have demonstrated that concomitant statin administration serves as an independent favorable prognostic factor, significantly improving both overall survival and progression-free survival in patients with advanced NSCLC and melanoma receiving PD-1/PD-L1 targeted therapies (156, 157). These clinical outcomes suggest that ATV is a highly viable adjuvant to mitigate immune evasion and enhance immunotherapy efficacy.

Combination with Metabolic Modulators: Population-based epidemiological studies reveal that the combination therapy of metformin and statins synergistically decreases the risk of HCC development in high-risk patients, demonstrating a clinical benefit that out-performs either monotherapy (158). To validate this metabolic synergy, prospective randomized controlled trials are investigating whether the combination of ATV and metformin can favorably alter tumor biology, metabolomics, and pathological variables in patients with clinically significant prostate cancer (159).

Combination with Chemoradiotherapy and Targeted Therapies: Beyond retrospective observations, prospective clinical trials are actively evaluating ATV combinations in solid tumors. For instance, a prospective Phase II clinical trial assessing ATV in combination with standard temozolomide and radiotherapy for newly diagnosed glioblastoma confirmed the safety and tolerability of this multimodality approach. Although ATV did not significantly improve progression-free or overall survival, the study identified high baseline LDL levels as an independent prognostic factor for poor cancer-related outcomes (160). Furthermore, in hormone-dependent malignancies, a prospective Phase II proof-of-concept trial investigated the combination of ATV with ADT in castration-resistant prostate cancer. Although the study did not meet its primary endpoint for significant PSA reduction, it provided early signs of disease stabilization, as evidenced by decreased PSA velocities in a subset of patients (78).

### 14.6 Future perspectives and pharmacological optimization

While ATV shows promising oncological potential, the high doses often required for anticancer efficacy *in vivo* can increase the risk of systemic toxicities. To improve its safety and pharmacological efficacy, future research should prioritize advanced delivery, precision biomarkers, and multimodal clinical approaches. First, employing tumor-targeted delivery systems, such as ATV-loaded micro-nanoparticles, can significantly improve intratumoral bioavailability and minimize off-target effects (150). Second, validating predictive biomarkers—such as specific lipid metabolism profiles or genetic vulnerabilities—is crucial for identifying patient subpopulations most likely to benefit from personalized ATV dosing (126). Finally, rationally designed combinations of ATV with immune checkpoint inhibitors or chemoradiotherapy can achieve effective synthetic lethality at lower, safer statin doses (157, 160). Integrating these pharmacological innovations will be essential for successfully translating ATV into a cancer therapeutic.

## 15 Conclusion

ATV demonstrates multifaceted anticancer activity by inhibiting the mevalonate pathway to impair tumor progression, metastasis, and immune evasion, while concurrently protecting normal tissues from chemoradiotherapy-induced injury. Not only have preclinical models been instrumental in elucidating these pleiotropic mechanisms, but the translational value of ATV is also now increasingly validated through clinical trials and real-world cohorts. To overcome the limitations of monotherapy and fully leverage its favorable safety and cost profile, future oncological applications should prioritize rationally designed combination regimens. By integrating ATV with immune checkpoint inhibitors, metabolic modulators, or conventional therapies, its dual capacity for tumor suppression and normal tissue protection can be effectively harnessed to improve survival outcomes in patients.
