# Supplementary figures and images for: Atorvastatin as a pleiotropic anticancer agent: mechanisms, evidence, and therapeutic repurposing potential
Source: Front Immunol. 2026 Apr 24;17:1808729. doi: 10.3389/fimmu.2026.1808729 (PMC13153102; doi:10.3389/fimmu.2026.1808729)

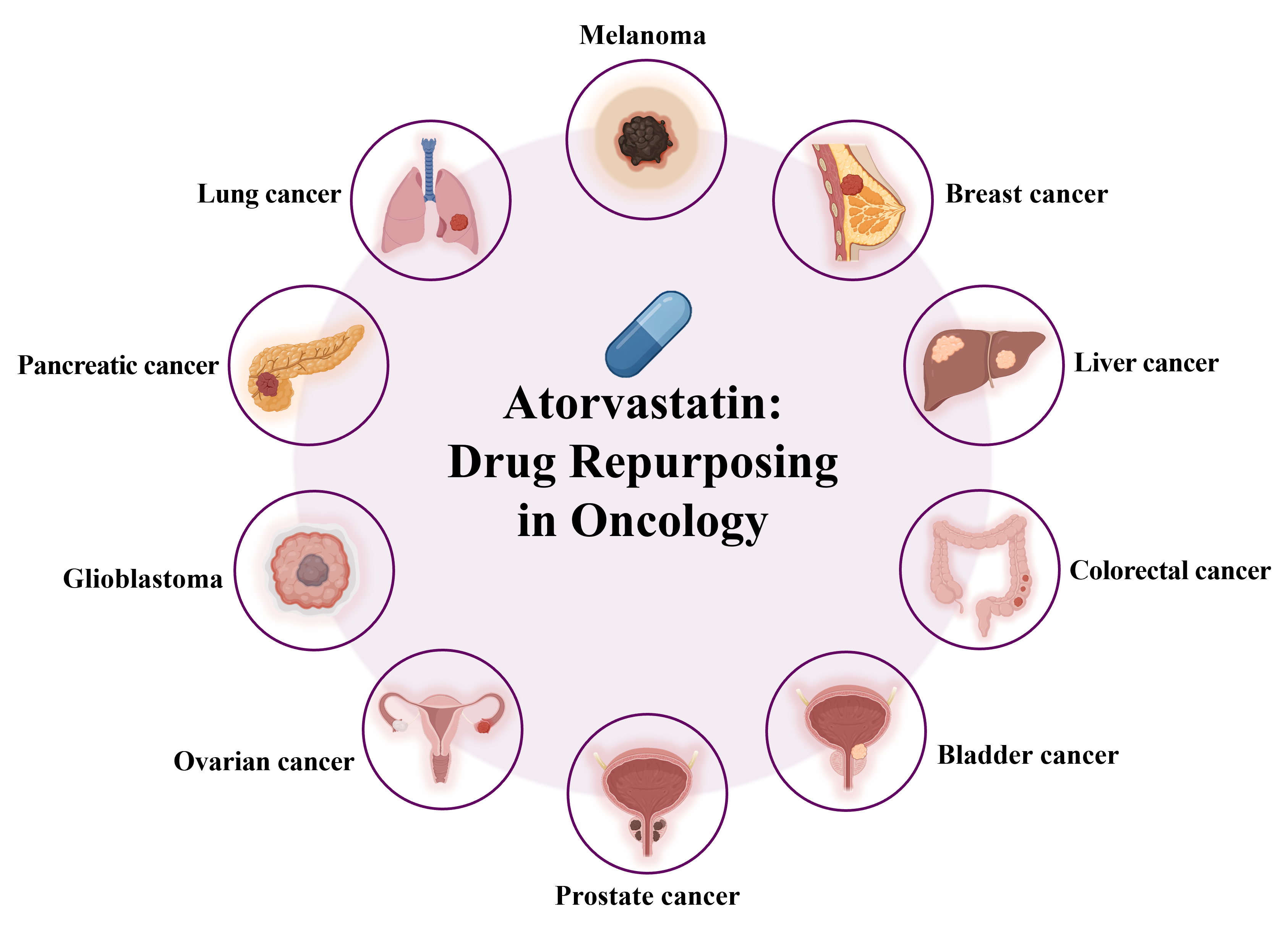

Supplement: Supplementary file 1 [file Image1.tif]
