# Supplementary material for: Atorvastatin as a pleiotropic anticancer agent: mechanisms, evidence, and therapeutic repurposing potential
Source: Front Immunol. 2026 Apr 24;17:1808729. doi: 10.3389/fimmu.2026.1808729 (PMC13153102; doi:10.3389/fimmu.2026.1808729)
Supplement: Supplementary file 2 [file Table1.docx]

Table 1. Summary of Preclinical Evidence (*In Vitro* and *In Vivo*) of Atorvastatin in Various Cancers

| **Cancer Type** | ***In Vitro* Models (Cell Lines)** | **Key *In Vitro* Mechanisms & Outcomes** | ***In Vivo* Models (Animal)** | **Key *In Vivo* Outcomes** |
| --- | --- | --- | --- | --- |
| Lung Cancer | A549 | G2/M arrest; apoptosis induction via Cav1 downregulation; suppression of Rac1/ROS/VEGF and MMP-9 ^[17, 40, 42]^. | BaP-induced lung cancer model | Attenuated tumor damage; upregulation of Bcl-2 and downregulation of Bax/caspase-3 ^[43]^. |
| Hepatocellular Carcinoma | HUH-7 | Antagonization of HBx-driven PI3K/Akt signaling; disruption of IL-6/STAT3 autocrine cascade; YAP1 downregulation ^[48, 50-52, 59]^. | MYC-driven, Aflatoxin-induced, and High-fat diet models | Delayed hepatocarcinogenesis; attenuated hepatic fibrosis; potential context-dependent promotion under high fatty acid diets ^[60-63, 142]^. |
| Breast Cancer | MDA-MB-231 (ER-), MCF-7 (ER+) | ER-: Inhibits GGTase and PBK, suppresses YAP. ER+: Concentration-dependent dual effect (protective autophagy vs. apoptosis) ^[20, 70-73]^. | Immunocompromised xenograft models | Attenuation of metastatic outgrowth; immunomodulation via PD-L1 downregulation ^[26]^. |
| Prostate Cancer | PC-3, LNCaP | Disruption of Ras/Rho geranylgeranylation; induction of cytoprotective autophagy; radiosensitization via HIF-1α/ROS modulation ^[28, 85-89]^. | Immunodeficient xenograft models | Synergistic tumor growth suppression when combined with celecoxib or radiotherapy ^[52]^. |
| Pancreatic Cancer | Pancreatic ductal adenocarcinoma (PDAC) lines | Inhibition of PI3K/AKT signaling; blockage of mutant p53 nuclear translocation ^[100]^. | p48Cre/+ LSL-KrasG12D/+ and p53 R172H models | Delayed progression from PanIN to PDAC; extended survival; synergistic efficacy with gemcitabine ^[96, 98-100]^. |
| Colorectal Cancer | HCT116, HT29 | Attenuation of COX-2/PGE2/β-catenin axis; concentration-dependent inhibition of angiogenesis ^[106]^. | BRAF-mutant, BVE, and Azoxymethane models | Normalized crypt cell density; cell cycle arrest and reduced overall tumorigenicity ^[107-109]^. |
| Bladder Cancer | T24, J82 | Triggering of autophagic flux; synergistic cholesterol suppression via AMPK/SREBP2 pathway ^[51, 113, 114]^. | BBN-induced rodent model | Significant reduction in bladder tumor incidence and volume ^[110]^. |
| Melanoma | A375 | Impairment of RhoC isoprenylation; potent inhibition of invasion and metastasis ^[115, 119, 120]^. | B16F10 or xenograft models | Inhibition of pulmonary colonization (extravasation and adhesion) ^[116]^. |
| Glioblastoma | Conventional and 3D glioma spheroids | Impaired EGFR/AKT signaling; downregulation of VEGF-A and CXCR4; disrupted lipid metabolism ^[124-128]^. | Glioma xenograft models | Suppressed gliomagenesis and angiogenesis; enhanced temozolomide efficacy ^[122, 129, 130]^. |
| Ovarian Cancer | Ovarian cancer cell lines | G1-phase arrest; ER stress and ROS overproduction; initiation of mitochondrial apoptosis;  Suppressed migratory and invasive capacities ^[136]^. | - | -. |

Table 2. Summary of Clinical Evidence and Translational Potential of Atorvastatin

| **Cancer Type** | **Study Type / Level of Evidence** | **Regimen / Combination** | **Key Clinical Outcomes** |
| --- | --- | --- | --- |
| Lung Cancer | Large-scale cohorts & real-world data | Monotherapy / Combination with Immune Checkpoint Inhibitors (ICIs) | Improved survival in advanced NSCLC [34, 41]; combination with PD-1/PD-L1 therapies improves OS and PFS ^[156, 157]^. |
| Melanoma | Real-world clinical analyses | Combination with Immune Checkpoint Inhibitors | Concomitant statin use acts as an independent favorable prognostic factor for OS and PFS ^[156, 157]^. |
| Hepatocellular Carcinoma | Population-based epidemiological studies | Combination with Metformin | Synergistic decrease in HCC incidence among high-risk patients compared to monotherapy ^[158]^. |
| Prostate Cancer | Database analyses & Prospective Phase II/ RCT | Monotherapy / Combination with Metformin or Androgen Deprivation Therapy | Dose-dependent reduction in biochemical recurrence post-prostatectomy ^[79-81]^; provided early signs of disease stabilization (decreased PSA velocities) in castration-resistant prostate cancer ^[78]^. |
| Pancreatic Cancer | Clinical cohorts | Monotherapy | Statin use post-diagnosis is associated with reduced mortality in pancreatic cancer patients ^[95]^. |
| Glioblastoma | Prospective Phase II Trial | Combination with standard Temozolomide and Radiotherapy | Safety and tolerability confirmed; while it did not significantly improve PFS/OS, high baseline LDL levels were identified as an independent prognostic factor for poor outcomes ^[160]^. |
| Multiple Cancers | Epidemiological & Real-world cohorts | Combination with Ezetimibe | Dual metabolic targeting provides synergistic reduction in overall cancer risk compared to statin monotherapy ^[30, 32]^. |
